# Intensive care management of patients with acute intermittent porphyria: Clinical report of four cases and review of literature

**DOI:** 10.4103/0972-5229.68222

**Published:** 2010

**Authors:** Madhur Mehta, Girija P. Rath, Uma P. Padhy, Manish Marda, Charu Mahajan, Hari H. Dash

**Affiliations:** **From:** Department of Neuroanaesthesiology, All India Institute of Medical Sciences, New Delhi, India; 1 Department of Neurology, All India Institute of Medical Sciences, New Delhi, India

**Keywords:** Acute intermittent porphyria, intensive care management, respiratory failure

## Abstract

Acute intermittent porphyria (AIP), the most common and the most severe form of acute hepatic porphyria, is an autosomal dominant condition. It results from lower-than-normal levels (less than 50%) of porphobilinogen (PBG) deaminase. Patients may present commonly with gastrointestinal complaints and neuropsychiatric manifestations. Diagnosis may be confirmed with the presence of intermediary metabolites of haem synthesis, amino levulinic acid (ALA) and PBG in urine or with specific enzyme assays. Abdominal pain is the most common symptom (90%). Peripheral polyneuropathy, primarily motor with flaccid paresis of proximal musculature, with or without autonomic involvement, is characteristic. Respiratory failure necessitates ventilator and intensive care support. Avoidance of precipitating factors and the use of haem preparations and intravenous dextrose form the basis of management. Gabapentin and propofol, rather than the conventional antiepileptics appear to be the appropriate choice for seizure control. Here, we present intensive care management of four cases of AIP with varying clinical presentation.

## Introduction

Acute intermittent porphyria (AIP) is an autosomal dominant inborn error characterized by decreased activity of porphobilinogen (PBG) deaminase (also known as hydroxymethylbilane synthase or uroporphyrinogen I synthetase), leading to increased levels of haem precursors, namely amino levulinic acid (ALA) and PBG.[1] It is the commonest and most severe form acute porphyria. Abdominal pain, neurological dysfunction and psychiatric disturbances form the classic triad of AIP. However, presentation like respiratory failure necessitates admission to intensive care unit (ICU). Management of such patients during the crisis period has rarely been discussed.[2] Here, we report 4 cases of AIP admitted over a period of 2 years, in our ICU. Various clinical presentations and relevant issues pertaining to intensive care management in these patients have been reviewed.

## Case Report [Table 1]

### Case 1

A 26-year-old male was admitted with complaints of weakness, fever, generalized tonic clonic seizures (GTCS), and urinary incontinence for a month. GTCS episodes were accompanied by transient loss of consciousness (LOC) and frothing from the mouth, since last 3 days. On examination, deep tendon reflexes (DTR) were absent, and plantar response was flexor. Facial muscle weakness was present, while other cranial nerves were normal. His respiratory rate (RR) was 14/min, heart rate (HR) 88/min, and blood pressure (BP) was 138/98 mmHg. Intravenous sodium valproate was started for control of seizures. On the 18^th^ day of admission, he aspirated following an episode of GTCS. Trachea was intubated and he was put on mechanical ventilation. Investigations were within normal limits except for a positive urine PBG. A diagnosis of acute porphyria was made. He was managed conservatively on a high carbohydrate diet and broad spectrum antibiotics (c-bactam and metrogyl). Sodium valproate was replaced with gabapentin. Subsequently, he developed severe pulmonary infection and tracheostomy was done on the 28^th^ day. Severe sepsis associated hypotension and tachycardia during the later course persisted despite noradrenaline infusion, the patient deteriorated neurologically and died on the 30^th^ day of admission.

**Table 1:** Patient presentation, course of illness, management and outcome

Age/sex	Presented with	Diagnosis	Reason for mechanical ventilation	Associated problems	Management	Anti-epileptic drugs used	Course	Outcome/Discharge
26/Male	Fever weakness urine incontinence GTCS, LOC	Urine porphobilinogen	Aspiration following an episode of GTCS		High carbohydrate diet antibiotics	Sodium valproate gabapentin	Severe pulmonary infection leading to sepsis tracheostomy	Died on 30^th^ day of admission
13/Male	Acute abdomen, status epilepticus, altered sensorium generalized, hypotonia	Urine porphyrinogen	Low GCS	Recurrent seizures on multiple AEDs, altered (hyperactive) behaviour, poor performance	Propofol infusion high carbohydrate diet antibiotics	Leviteracetum Gabapentin	Improved over 1 week trachea extubated	Discharged on 9^th^ day
19/Male	Ascending weakness all 4 limbs, swallowing difficulty, acute abdomen, GTCS	Electrophysiological study: asymmetric sensory motor, mixed axonal, demyelinating neuropathy Urine porphyrinogen	Respiratory distress	Tuberculosis received antitubercular medication	High carbohydrate diet antibiotics	Phenytoin Sodium Sodium Valproate Leviteracetum Gabapentin	Tracheostomied Improved swallowing and neurological status over 3 week Weaned of from ventilator Partial recovery of limbs	Disharged on 27^th^ day of admission Motor power: 3/5
22/Male	Abdominal pain vomiting constipation, repeated GTCS	Electrophysiological study: asynnetric sensori-motor mixed neuropathy Urine porphyrinogen	Poor cough reflex, respiratory distress	Quadriparesis Bulbar palsy Autonomic dysfunction	High carbohydrate diet Antibiotics	Leviteracetum Gabapentin	Require metoprolol and enalapril for control of haemodynamics Improved bulbar function Stayed in ICU for 3weeks	Discharged on 30^th^ day with an advice for follow-up

GTCS: Generalized tonic clonic seizures, LOC: Loss of consciousness, GCS: Gasgow coma scale

### Case 2

A 13-year-old male child with history of recurrent seizures was admitted with complaints of acute abdominal pain, altered behavior, and development of status epilepticus. The child had a history of GTCS with LOC at 2 years of age which was uncontrolled despite escalating doses of anti-epileptic drugs (AEDs). He was dropped out of school due to hyperactive behavior and poor performance. He underwent a negative laparotomy for acute abdominal pain two years before. On examination, RR was 18/min and regular; HR was 84/min and BP 100/70 mmHg. He had altered sensorium (GCS = E_1_ V_1_ M_3_), with generalized hypotonia (motor power = 1-2/5). Bilateral plantar responses were flexor. A magnetic resonance image (MRI) of the head revealed no other abnormality. As the urine porphyrinogen was found to be positive; a diagnosis of acute porphyric crises was established. He required tracheal intubation in view of a low GCS, and was started on propofol infusion for sedation. Initially, leviteracetam and later, gabapentin was given as a part of medical management. The hemodynamic parameters remained stable and the neurological condition improved over a period of one week. Propofol was discontinued, and the patient was weaned off from the ventilator. Trachea was extubated on the 7^th^ day of admission and the patient was discharged after another two days of observation with an advice for follow-up.

### Case 3

A 19-year-old male was admitted with ascending weakness of all four limbs, which developed over a week. He had difficulty in swallowing and a nasal twang to his voice. He had an episode of severe abdominal pain with vomiting, followed by generalized seizures. He was treated with phenytoin initially, followed by sodium valproate, which resulted in worsening of the condition. He sufferred a similar attack of GTCS, severe abdominal pain and vomiting with mesenteric lymphadenopathy, pleural effusion and ascites, one year back for which he received anti-tubercular medication for six months (ATT). On examination, the patient had a HR= 82/min and BP = 110/70 mmHg. He was conscious, alert but quadriparetic (motor power = 0/5) with bifacial and bulbar weakness. Bilateral plantar responses were flexor. As he developed respiratory distress, trachea was intubated, and he was put on ventilatory support. Rest of the examination was within normal limits. Urine porphyrinogen was positive and hence, a diagnosis of acute porphyria was made. Electrophysiological study revealed asymmetric sensorimotor, mixed axonal and demyelinating neuropathy involving all limbs, predominantly the upper limbs. Initially, he was treated with leviteracetam 500 mg twice daily, for seizure control; later, gabapentin 300 mg twice daily was given as urine porphyrinogen increased with the use of the former drug. Conservative management was continued in the ICU for 3 weeks. During this period, he was tracheostomized, and gradually, weaned off from ventilatory support. The patient was discharged after neurological improvement was observed in the form of return of bulbar functions such as swallowing, and partial recovery of limbs (motor power of 2-3/5).

### Case 4

A 22-yr-old male was admitted to our ICU with complaints of abdominal pain, vomiting, constipation, and repeated episodes of GTCS for 1 month. He developed quadriparesis, predominantly of the proximal musculature for four days and difficulty in speaking and swallowing for two days. On examination, the patient had flaccid quadriparesis (motor power 0-1/5) with bulbar and bifacial paralysis. DTRs were absent and plantar response was flexor. BP was 170/118 mm Hg and HR was 118/min. In view of a poor cough reflex and respiratory effort, the patient was put on ventilatory support after tracheal intubation. Urine porphyrinogen was tested as positive and acute porphyria diagnosed. Electrophysiological studies revealed asymmetric sensorimotor mixed neuropathy. In addition to high carbohydrate, antibiotics, gabapentin and leviteracetam, the patient required metoprolol and enalapril for control of hemodynamics. The patient stayed in the ICU for a period of 20 days during his condition was improved, and he was weaned off from the ventilator. Neurological improvement was observed in the form of return of bulbar functions and partial recovery of limbs (motor power of 2-3/5). The patient was discharged from the hospital on the 30^th^ day, with an advice for follow-up.

## Discussion

Acute porphyrias (AIP, hereditary coproporphyria, variegate porphyria and ALA dehydratase deficient porphyria) are characteristically hepatic porphyria, presenting with neurological manifestations. AIP usually presents with neurovisceral and psychiatric disturbances like abdominal pain (90% of patients), constipation, insomnia, depression, disorientation, and hallucinations. In acute porphyric crisis, encephalopathy varying from confusion to frank psychosis can occur concomitantly with hypothalamic involvement and metabolic derangement of inappropriate secretions of ADH (anti-diuretic hormone).[2] Generalized seizures, myoclonic activity or coma may be observed due to neurological effects or hyponatremia.[3]

Polyneuropathy and painful flaccid paralysis predominantly involve upper limbs; preferentially affecting the proximal musculature with occasional sensory involvement. Motor weakness may be asymmetric and focal. Cranial nerves may be involved. Progressive muscle weakness can lead to life threatening respiratory and bulbar paralysis.

Autonomic disturbances may manifest as urinary retention, paralytic ileus, restlessness, tremors, excessive sweating, tachycardia and fluctuating blood pressure, typically labile hypertension.[4] Sometimes, persistent hypotension may require inotropic support. Complications like bradycardia and sudden cardiac arrest have also been reported.[1]

The reason for neurological involvement in acute porphyrias remains poorly understood. Direct neurotoxicity of delta-ALA by interaction with GABA receptor, altered tryptophan metabolism, or a neural respiratory haem-dependent enzymatic deficiency in nerve cells has been hypothesized.[5] Nevertheless, axonal degeneration of peripheral and autonomic nerve fibres rather than demyelination seems to be responsible. The neurological effects of acute porphyria are generally reversible, though incomplete recovery and residual paresis have also been reported. The reported mortality in porphyric polyneuropathy varies from 20-50%.[2] Sudden cardiac arrest secondary to autonomic dysfunction is known to cause death in these patients.

Diagnosis of AIP requires a clinical suspicion with documentation of ALA and PBG in freshly voided urine. Classic burgundy red discoloration of long stored urine or Watson-Schwartz test using Ehrlich’s aldehyde reagent are useful for screening. Quantitative measurements of PBG and ALA in urine or erythrocyte hydroxymethylbilane synthase enzyme test are more reliable confirmatory tests. These tests are suitable for screening asymptomatic family members, but, exorbitant cost limits their routine use. AIP at times mimic Guillain Barre Syndrome (GBS).[6] Absence of lymphocytosis with raised protein content in CSF favors a diagnosis of GBS rather than AIP.

Patients of AIP may require ventilatory support for progressive ascending paralysis with respiratory and/or bulbar muscle involvement. Varying neurological and mental dysfunction may necessitate treatment with analgesics, antiepileptics, and sedatives in addition to general care. During acute attacks, narcotic analgesics may be required for abdominal pain while phenothiazines may be useful for nausea, vomiting, anxiety and restlessness. Propofol has been tried successfully as a sedative[7] as well as an antiepileptic,[8] particularly in ICU settings. The primary AEDs such as phenytoin, barbiturates, carbamazepine, and sodium valproate are unsafe and better avoided, in patients with seizures. Magnesium sulfate is useful for emergency treatment of seizures, but, not for long-term use. Gabapentin appears to be a safe and effective alternative with a promising future.[9] It is not appreciably metabolized in the liver nor does it have any effect on hepatic microsomal enzymes.

Haem in the form of hematin (Abott laboratories), haem albumin or haem arginate 3-4 mg/kg/day (Leiras Oy, Turku, Finland) infused daily for 3-4 days is the treatment of choice for AIP.[1] Haem acts by repressing the ALA synthetase enzyme and thus, further suppressing the production of haem precursors. Associated high cost and lack of availability are the main drawbacks. It was not used in our patients due to unavailability. Alternatively, carbohydrate rich diet is used. Intravenous dextrose in higher doses (300-500 gm/day) blocks induction of the enzyme and prevents accumulation of precursors. High-dose of dextrose may not only be used as a substitution; but also for providing a more complete parenteral nutritional regimen whenever oral feeding is delayed.

The most important step in preventing further crises is to withdraw the precipitating factors such as prolonged fasting, dehydration, stress, hypothermia, infection, and fever. Porphyrogenic drugs like barbiturate, antiepileptics (some), erythromycin, chloramphenicol etc. should be avoided.[1] These are inducers of hepatic metabolism thereby subjecting the patients to acute porphyric attacks. Hence, drugs not dependent on liver for metabolism should be preferred. Pregnancy, alcohol and female sex hormones are some other associated risk factors for acute crises.

To conclude, patients with AIP constitute a group of critically ill patients who require specialized care. High clinical suspicion, early diagnosis, and management of an acute attack along with measures to prevent future attacks are the mainstay of a favorable outcome. AIP should always be suspected in the setting of neuro-psychiatric manifestations in patients with gastrointestinal complaints. Families of these individuals should be subjected to suitable enzyme tests, where facilities exist, to screen asymptomatic relatives.
